# Supplementary material for: Viral Microglia Reprogramming Clears Oligomeric Neurotoxic Debris
Source: bioRxiv. 2026 Apr 8:2026.04.06.716590. Preprint. [Version 1] doi: 10.64898/2026.04.06.716590 (PMC13082030; doi:10.64898/2026.04.06.716590)
Supplement: Supplement 1 [file media-1.pdf]

SUPPLEMENTAL MATERIAL

Carter *et al*.
MDA5-mediated microglia reprogramming

Viral Microglia Reprogramming Clears Oligomeric Neurotoxic Debris

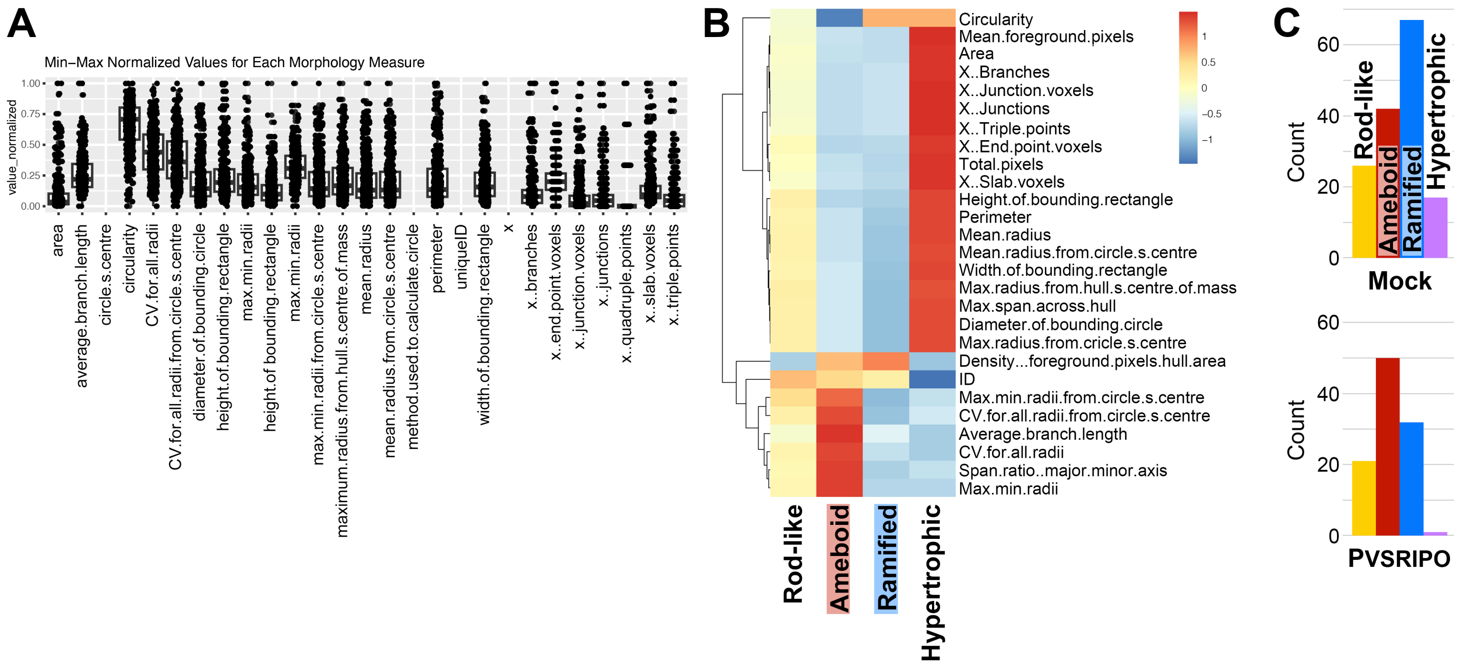

Figure S1 (Related to Figure 1C). Validating the microglia morphology analyses tool reported by Kim *et al*. (2024) for our approach. Extended data from analyses of a set of “training” images stained for Iba1 using MicrogliaMorphology (ImageJ) and MicrogliaMorphologyR (R package) toolsets. **(A)** Distribution of 27 distinct morphological features found in cell shapes from Images shown in Figure 1C [(Mock-treated) glioma-bearing brain, (PVSRIPO-treated) glioma-bearing brain were combined]. **(B)** MicrogliaMorphology-assigned clusters based on the defined 27 features of Iba1-stained phagocytes in sections shown in Figure 1C. **(C)** Preliminary analyses of changes in cluster frequency in Mock- vs. PVSRIPO-treated glioma-bearing brain, based on validation experiments with Iba1-stained samples.

Table S1 (Related to Figures 3 and 4; attached separately). Conserved markers across treatments for astrocyte (Astrocyte _markers), neoplastic (Cancer_markers), bone marrow-derived myeloid cells (Macrophage_ markers), microglia (Microglia_markers), neurons (Neuron_markers), and pericyte & fibroblast (Pericyte_Fibroblast_markers) clusters; differentially expressed genes conserved across treatment comparing microglia to bone marrow-derived myeloid cells (Microglia_v_ Macrophages _markers); treatment markers within microglia comparing PVSRIPO to mock (RIPO_v_Mock_microglia).

Antigen	Fluorophore	Vendor	Cat #	Dilution	RRID
Mouse:					
CD45	BUV395	BD	564279	100x	AB_2651134
CD3	PE	Biolegend	100206	100x	AB_312663
IA/IE	BV785	Biolegend	107645	100x	AB_2565977
CD11c	APC	Biolegend	117310	100x	AB_313779
CD11c	APC/Cy7	Biolegend	117324	100x	AB_830649
CD11c	BV421	Biolegend	117330	100x	AB_11219593
F4/80	BUV805	BD	749282	100x	AB_2873657
IA/IE	BV711	Biolegend	107643	100x	AB_2565976
F4/80	BV605	Biolegend	123133	100x	AB_2562305
CD11b	BV711	Biolegend	101236	100x	AB_11203704
Ly6C	PerCP-Cy5.5	BD	560525	100x	AB_1727558
F4/80	PE-Cy5	Biolegend	123112	100x	AB_893482
Ly6G	BV605	Biolegend	127639	100x	AB_2565880
Siglec-F	PE-Cy7	Biolegend	155528	100x	AB_2890715
CD3	BV605	Biolegend	317322	100x	AB_2561911
CD4	FITC	Biolegend	100406	100x	AB_312691
H-2KB/D	PE	Biolegend	114608	100x	AB_313599
CD45	BUV737	Biolegend	612778	100x	AB_2870107
CD86	FITC	Biolegend	105006	100x	AB_313149
CD68	APC/Fire-750	Biolegend	137042	100x	AB_2910295
H2KB	PE-Cy7	Invitrogen	25-5958-82	100x	AB_2573505
Cx3cr1	PerCP-Cy5.5	Biolegend	149010	100x	AB_2564494
CD80	PE-Cy7	Biolegend	104734	100x	AB_2563113
Ki-67	BV421	Biolegend	652411	100x	AB_2562663
Tmem119	PE-Cy7	Invitrogen	25-6119-82	100x	AB_2848312
CD68	FITC	Biolegend	137006	100x	AB_10578412
TREM2	FITC	Invitrogen	MA5-28223	100x	AB_2745193
CD40	PE-594	Biolegend	124630	100x	AB_2572185
H2KB: SIINFEKL	PE	Biolegend	141604	100x	AB_10895905

Antigen	Fluorophore	Vendor	Cat #	Dilution	RRID
Human:					
CD11b	APC	Biolegend	982604	200x	AB_2632619
CD11c	BUV395	BD	563787	200x	AB_2744274
CD155	PE	Biolegend	337610	200x	AB_2174019
HLADR	BV786	Biolegend	307642	200x	AB_2563461
CD68	APC/Fire-750	Biolegend	333824	200x	AB_2800878
Tmem119	Alexa 647	Biolegend	853312	200x	AB_3097501
Cx3cr1	BV785	Biolegend	341628	200x	AB_2810535
MERTK	PE-Cy7	Biolegend	367610	200x	AB_2687287
IF (mouse):					
Iba1		CST	17198		AB_2820254
Tmem119		CST (E3E10)	90840		AB_2928137
ISG15		Invitrogen	703132		AB_2784563
A11		Thermo F.	AHB0052		AB_10376183

Table S2. Antibodies used in this study.
